# The Impact of Poor Nonverbal Social Perception on Functional Capacity in Schizophrenia

**DOI:** 10.3389/fpsyg.2022.804093

**Published:** 2022-02-23

**Authors:** Victoria Chapellier, Anastasia Pavlidou, Lydia Maderthaner, Sofie von Känel, Sebastian Walther

**Affiliations:** Translational Research Center, University Hospital of Psychiatry and Psychotherapy, University of Bern, Bern, Switzerland

**Keywords:** communication, gesture, social cognition, functional domains, affect, social functioning, psychosis

## Abstract

**Background:**

Nonverbal social perception is the ability to interpret the intentions and dispositions of others by evaluating cues such as facial expressions, body movements, and emotional prosody. Nonverbal social perception plays a key role in social cognition and is fundamental for successful social interactions. Patients with schizophrenia have severe impairments in nonverbal social perception leading to social isolation and withdrawal. Collectively, these aforementioned deficits affect patients’ quality of life. Here, we compare nonverbal social perception in patients with schizophrenia and controls and examine how nonverbal social perception relates to daily functioning.

**Methods:**

We compared nonverbal social perception in 41 stable outpatients with schizophrenia and 30 healthy controls using the Mini Profile of Nonverbal Sensitivity (Mini-PONS). The participants evaluated 64 video clips showing a female actor demonstrating various nonverbal social cues. Participants were asked to choose one of two options that best described the observed scenario. We correlated clinical ratings (Positive and Negative Syndrome Scale, Brief Negative Syndrome Scale), Self-report of Negative Symptoms, and functional assessments (functional capacity and functional outcome) with Mini-PONS scores.

**Results:**

Patients performed significantly poorer in the Mini-PONS compared to controls, suggesting deficits in nonverbal social perception. These deficits were not associated with either positive symptoms or negative symptoms (including self-report). However, impaired nonverbal social perception correlated with distinctive domains of BNSS (mainly avolition and blunted affect), as well as functional capacity and functional outcome in patients.

**Conclusion:**

We demonstrate that nonverbal social perception is impaired in stable outpatients with schizophrenia. Nonverbal social perception is directly related to specific negative symptom domains, functional capacity and functional outcome. These findings underline the importance of nonverbal social perception for patients’ everyday life and call for novel therapeutic approaches to alleviate nonverbal social perception deficits.

## Introduction

Schizophrenia is a severe and debilitating psychiatric disorder that affects nearly 1% of the world’s population (McGrath et al., 2008). Schizophrenia is characterized by delusions, hallucinations, negative symptoms, disorganization symptoms according to DSM-5, including impaired social cognition (Goldsmith and McFall, 1975; Green et al., 2008a,^2019^). Generally, social cognition refers to psychological processes that allow us to decode the behaviors and intentions of others (Frith and Frith, 2007; Adolphs, 2009). Impaired social cognition is not only prevalent in chronic schizophrenia patients, but also in early-onset psychosis and early psychosis patients (Fornells-Ambrojo and Garety, 2009; Barkl et al., 2014; Giannitelli et al., 2015; Healey et al., 2016). Therefore, these deficits occur across different stages of the disorder. Social cognitive deficits are assumed to be a stable trait that precedes and predicts the onset of schizophrenia (Frith and Corcoran, 1996; Riveros et al., 2010; Savla et al., 2013; Pinkham et al., 2014; Green et al., 2015), and informs on the frequency of patients’ relapse (Mcglashan, 1986). In schizophrenia, social cognition acts as a mediator between neurocognition and functional outcome (Vauth et al., 2004; Brekke et al., 2005; Couture et al., 2006; Sparks et al., 2010; Mancuso et al., 2011; Galderisi et al., 2020). Hence, these impairments play a key role not only in the development of the disorder, but also in the functional outcome of patients. Social cognitive impairment in schizophrenia encompasses multiple domains.

In schizophrenia, all of the five well-established social cognitive domains are impaired (Pinkham et al., 2014): social perception (Toomey et al., 2002), social knowledge (Penn et al., 2002), emotion processing (Pollard et al., 1995), attributional processing (Green and Horan, 2010), and theory of mind (Greig et al., 2004; Green and Horan, 2010). A meta-analysis revealed that social perception and theory of mind were most severely impaired in schizophrenia (Savla et al., 2013). The majority of previous studies focused on theory of mind and emotion processing. However, only a few studies focused on the nonverbal aspects of social perception (emotional prosody, facial expressions, body movements) in schizophrenia and used heterogeneous tasks (Pinkham et al., 2017).

Nonverbal social perception, which is the ability to decode relevant nonverbal interpersonal cues (Morrison and Bellack, 1981; Toomey et al., 2002) is impaired in schizophrenia patients (Walther et al., 2015). For example, schizophrenia patients exhibit decreased ability to evaluate nonverbal cues such as prosody (Murphy and Cutting, 1990), facial expressions (Cutting, 1981; Colussy and Zuroff, 1985; Borod et al., 1990; Edwards et al., 2002; van t’wout et al., 2007; McIntosh and Park, 2014), as well as, hand and body movements (Monti and Fingeret, 1987; Toomey et al., 2002; Goldin-Meadow and Alibali, 2013; Walther et al., 2015). Incorrect interpretation of facial expressions and body movements limits schizophrenia patients’ communication: they tend to perceive ambiguous gestures and direct gaze as self-referential or threatening (Bucci et al., 2008; White et al., 2016; Wastler and Lenzenweger, 2018). In addition, impaired gesture perception has been linked to deficits in domains of visual information processing (Matthews et al., 2013; Millman et al., 2014; Walther et al., 2015; Gupta et al., 2021), which affects patients’ attention and working memory (Green et al., 2008b; Jahshan et al., 2012). In schizophrenia, associations between the ability to evaluate nonverbal cues and symptoms are unclear (Toomey et al., 2002; Walther et al., 2015, 2016).

Despite the progress made in understanding social cognitive deficits in schizophrenia, the role of nonverbal social perception in schizophrenia remains poorly understood (Green et al., 2005). While in most reports associations between poor nonverbal social perception and positive symptoms (Toomey et al., 2002; Giannitelli et al., 2015) as well as negative symptoms are lacking (Olbert et al., 2013; Walther et al., 2016), others report the opposite (Walther et al., 2015). Therefore, the relationship between nonverbal social perception and symptoms remains unresolved. However, impaired nonverbal social perception are assumed to be associated with disorganization symptoms (Toomey et al., 2002), which have shown to mediate between nonverbal social perception deficits and poor functioning in schizophrenia (Engelstad et al., 2017). In fact, nonverbal social perception deficits may have an important impact on schizophrenia patients’ social interaction, as these deficits are linked to patients’ gesture performance (Walther et al., 2015) and overall functioning (Morrison et al., 1988; Hooker and Park, 2002; Sergi et al., 2006; Walther et al., 2016).

In patients with schizophrenia, deficits in nonverbal social perception have been associated with poor functional outcome (Morrison et al., 1988; Hooker and Park, 2002; Sergi et al., 2006) and functional capacity (Walther et al., 2016). In general, functional capacity refers to the relevant real-world adaptive skills for daily functioning, such as managing finances or scheduling an appointment (Patterson et al., 2001). Nonverbal social perception deficits in schizophrenia have been shown to predict functional capacity and outcome at 6 months follow-up in schizophrenia patients (Walther et al., 2016). Consequently, nonverbal social perception is closely linked to overall functioning, and therefore should be a target for pharmacological and non-pharmacological interventions. While the association between the ability to decode facial expressions and functional capacity has intensively been investigated in schizophrenia (Olbert et al., 2013; Abram et al., 2014), studies investigating the link between nonverbal social perception as a whole (including body movements and emotional prosody perception) and functional capacity are scarce.

This study aimed at determining whether schizophrenia outpatients perform poorer in nonverbal social perception than healthy controls. We hypothesized that poor nonverbal social perception is linked to limited functional capacity and poor functional outcome. We expect nonverbal social perception to have different effects on different functional domains. Hence, we explored six functional domains separately: physical functioning, personal care skills, interpersonal relationships, social acceptability, activities and work skills. In addition, we investigated the impact of symptoms such as positive symptoms, negative symptoms (including the domains of anhedonia, asociality, avolition, blunted affect, and alogia, as well as the item gesture expression) and the severity of symptoms on nonverbal social perception.

## Materials and Methods

In total, 41 schizophrenia patients (mean age = 38.6 years, SD = 12.2; 51.2% male) and 30 healthy controls (mean age = 40.0 years, SD = 12.2; 50.0% male) were included in this study (Table 1). We recruited patients with a diagnosis of the schizophrenia spectrum (33 patients with schizophrenia and 8 patients with the schizoaffective disorder) according to DSM-5 at the outpatient clinics of the University Hospital of Psychiatry and Psychotherapy, Bern. The recruitment period of participants started in December 2019 and ended in June 2021. This study focuses on baseline data of a larger project (Brain Stimulation And Group Therapy to Improve Gesture and Social Skills in Psychosis trial, clinicaltrials.gov NCT04106427). All patients consented to participate in the entire interventional study, were symptomatically stable and all but three received antipsychotic treatment. Healthy control participants were recruited by word-of-mouth, through leaflets at public places and a post on the website of the University Hospital of Psychiatry and Psychotherapy. For every participant, we acquired all baseline data within 2 days. All participants met the following criteria: 18–65 years of age, ability to provide written informed consent as documented by signature, no substance abuse or dependence other than nicotine, no past or current medical or neurological condition associated with impaired or aberrant movement and no epilepsy. In addition, controls had no history of any psychiatric disorder or first-degree relatives with schizophrenia spectrum disorders.

**TABLE 1:** Demographic and clinical characteristics.

	Patients (*N* = 41)	Controls (*N* = 30)	Comparison
**Demographics**			
Age (years)	38.6 ± 12.2	40.0 ± 12.2	*t* = 0.5; *p* = 0.651
Gender (%female)	51.2%	50.0%	
Education (years)	14.5 ± 3.3	16.2 ± 2.6	*t* = 2.4; *p* = 0.021*
Digit span backwards	4.4 ± 1.0	4.6 ± 1.1	*t* = 0.9; *p* = 0.371
Medication (CPZ-eq in mg)	455.0 ± 417.3		
**Assessments**			
PANSS positive^a^	12.4 ± 6.1		
PANSS negative^a^	17.3 ± 7.9		
PANSS total^a^	60.9 ± 21.5		
BNSS^a^	27.5 ± 15.6		
SNS	15.1 ± 6.7	4.3 ± 2.9	*t* = −9.1; *p* < 0.001***
Mini-PONS	43.6 ± 5.3	47.6 ± 4.4	*t* = 3.5; *p* < 0.001***
UPSA-brief^b^	83.8 ± 11.0	90.5 ± 7.4	*t* = 3.0; *p* = 0.003**
SLOF	183.5 ± 18.5	213.3 ± 4.0	*t* = 10.2; *p* < 0.001***

PANSS, positive and negative syndrome scale; BNSS, brief negative symptom scale; SNS, self-evaluation of negative symptoms; Mini-PONS, mini profile of nonverbal sensitivity; UPSA-brief, University of California San Diego performance-based assessment; SLOF, specific levels of functioning scale; Values represent the mean ± SD for each group. ^a^ was not assessed in healthy controls, ^b^ one missing value for one patient; * denotes a significant difference p-value < 0.05; ** denotes a significant difference p-value < 0.01, *** denotes significant differences p-value < 0.001. P-values marked with bold indicate statistically significant differences between the groups.

### Assessments

#### Behavioral Assessment

Nonverbal social perception was assessed with the Mini Profile of Nonverbal Sensitivity (Mini-PONS), which includes 64 videos (2 s each) showing a Caucasian woman (Rosenthal, 1980; Banziger et al., 2011). The Mini-PONS has four subscales (see Table 2): voice recordings/emotional prosody only (either content-filtered speech, or randomized splice speech), body movements only, facial expressions only, as well as, combined emotional prosody (content-filtered speech and randomized splice speech) and facial expression. The test was administered on a computer. Participants were asked to choose one of two options that best described the observed scenario. The total score for Mini-PONS ranges from 0 to 64 and the four Mini-PONS subscores range from 0 to 16. The test takes around 13 min.

**TABLE 2:** Comparison of the four PONS subscales between patients and controls, whilst controlling for education and working memory.

	Patients (*N* = 41)	Controls (*N* = 30)	Comparison	Effect size
Emotional prosody (voice recordings)	10.3 ± 2.1	11.8 ± 2.0	*F*_(1,70)_ = 6.6, *p* = 0.012*	η_p_^2^ = 0.090
Body movements	10.7 ± 2.3	11.2 ± 2.0	*F*_(1,70)_ = 0.1, *p* = 0.706	η_p_^2^ = 0.002
Facial expression	11.3 ± 1.8	11.8 ± 1.6	*F*_(1,70)_ = 0.6, *p* = 0.453	η_p_^2^ = 0.008
Emotional prosody and facial expression	11.2 ± 2.4	12.8 ± 1.5	*F*_(1,70)_ = 5.7, *p* = 0.010*	η_p_^2^ = 0.095

Values represent the mean ± SD for each group; * denotes significant differences p-value < 0.05. P-values marked with bold indicate statistically significant differences between the groups.

#### Clinical and Functional Assessments

To assess the current psychopathology of patients, we used the Positive And Negative Syndrome Scale [PANSS; (Kay et al., 1987)] and the 13-item Brief Negative Symptom Scale [BNSS; (Strauss et al., 2012)]. In both patients and controls, we collected a self-report of negative symptoms: the Self-Evaluation of Negative Symptoms (SNS) scale, which includes 20 items with a 3-point Likert scale, total score ranging from 0 to 40 (Dollfus et al., 2016).

Functional capacity was assessed with the brief version of the University of California San Diego Performance-Based Assessment (UPSA-brief) (Patterson et al., 2001). The UPSA-brief evaluates a person’s ability in managing finances (e.g., count money change) and communicating with others (e.g., reschedule an appointment). Functional outcome was measured with the Specific Levels of Functioning Scale (SLOF), which is a 43-item interview-based instrument rated on a 5-point Likert scale by a psychiatrist. The SLOF total score ranges from 43 to 215. SLOF does not focus on items relevant to psychiatric symptoms, nor cognitive impairment, but assesses behaviors and abilities essential to function in the community (Schneider and Struening, 1983).

To ensure reliability, three MD clinical raters, all currently in psychiatric residency (LM, DA, DB), were trained by the principal investigator (SW). The interrater reliability resulted in a very good mean of 0.95.

### Data Analyses

Data were analyzed using SPSS (version 28) and R (version 3.6.1). For this report, we excluded four patients due to visual impairment or difficulties to understand the experimental task. We used two-sample *t*-tests to compare the demographic data, nonverbal social perception, clinical assessments and functional outcomes, available for both patients and controls. Our Mini-PONS total scores were normally distributed (W_(71)_ = 0.97, *p* = 0.105). However, since three of the four Mini-PONS sub scores were not normally distributed (W_(71)_ > 0.95, *p* < 0.014) we applied a square root transformation to the Mini-PONS sub scores. As years of education differed between patients and controls (*t* = 2.35; *p* = 0.021; see Table 1) and as working memory has been reported to affect nonverbal social perception (Walther et al., 2015), we used education and working memory as covariates in our main analyses. We ran a parametric ANCOVA comparing Mini-PONS total score in patients and controls, as well as a parametric MANCOVA to compare the four Mini-PONS subscales in both groups, whilst controlling for education and working memory. Effect sizes estimates were calculated for the parametric ANCOVA and MANCOVA analyses.

In patients, we ran partial correlations between Mini-PONS total score and psychopathology as well as functional outcomes, whilst controlling for medication (CPZ-equivalent in mg per day; Table 1). Here, we corrected for multiple comparisons using the false discovery rate (FDR). Additionally, we ran explorative correlations between Mini-PONS total score and five BNSS subscales (Alogia, Blunted Affect, Asociality, Avolition, and Anhedonia), one BNSS item (Gesture Expression) as well as six SLOF subscales (Physical functioning, Personal care skills, Interpersonal relationships, Social acceptability, Activities of community living, and Work skills) in patients, whilst controlling for medication.

## Results

### Comparison of Nonverbal Social Perception Between Patients and Controls

**FIGURE 1:**
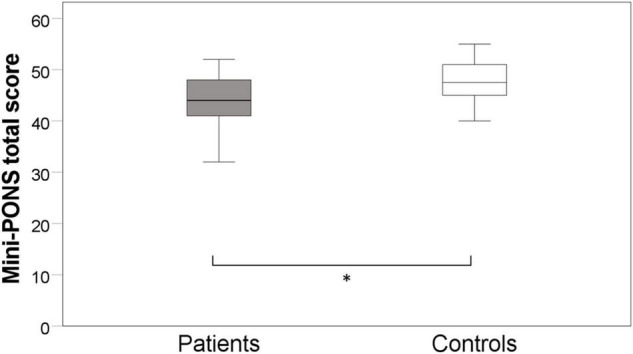
Group difference in nonverbal social perception. * denotes significant differences *p*-value < 0.05, whilst controlling for education and working memory.

**FIGURE 2:**
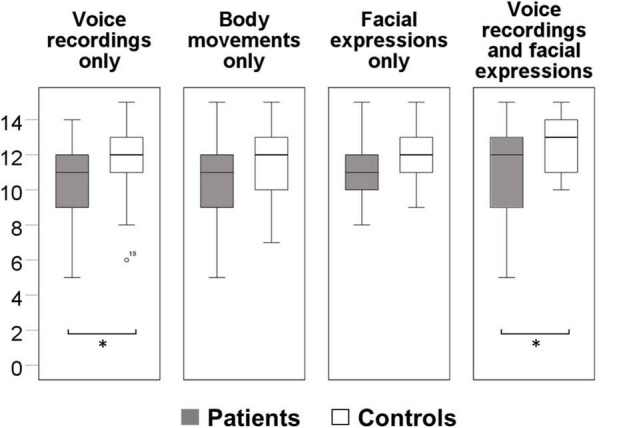
Group differences in emotional prosody, face and body movement perception. * denotes significant differences *p*-value < 0.05 whilst controlling for education and working memory.

Schizophrenia patients performed significantly poorer than controls (*F*_(1,70)_ = 7.1, *p* = 0.010, η_p_^2^ = 0.096) in the Mini-PONS when controlling for education and working memory, suggesting impairments in nonverbal social perception (see Figure 1). Regarding the four Mini-PONS sub scores, patients performed poorer in both scenarios with emotional prosody (voice recordings) only and emotional prosody combined with facial expressions, whilst controlling for education and working memory (see Table 2 and Figure 2). However, there was no significant group difference in videos with body movements only and facial expression only (see Table 2 and Figure 2). Hence, there was only a significant difference between patients and controls when emotional prosody was incorporated in the scenario. Following up on this group difference, we explored a group × stimulus type interaction in an additional 2×2 ANCOVA controlling for education and working memory. While we found main effects for group (controls > patients) and for stimulus type (bimodal > unimodal), we failed to detect an interaction of the two factors [(*F*_(1,70)_ = 0.1, *p* = 0.760, η_p_2 = 0.001)]; i.e., both groups improve with multimodal stimuli at comparable magnitude.

### Correlation Between Nonverbal Social Perception and Clinical Assessments/Functional Outcomes

**FIGURE 3:**
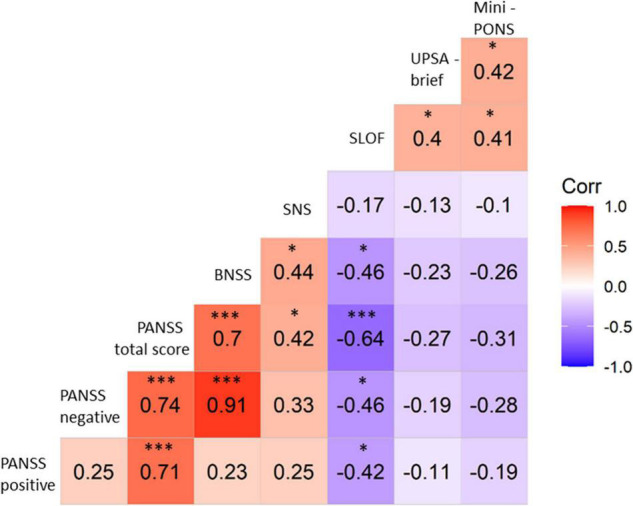
Correlations between Mini-PONS total score and clinical/functional tests controlling for medication. PANSS, positive and negative syndrome scale; BNSS, brief negative symptom scale; SNS, self-evaluation of negative symptoms; SLOF, specific levels of functioning scale; UPSA-brief, University of California San Diego performance-based assessment; Mini-PONS, mini profile of nonverbal sensitivity; * denotes significant correlations *p*-value < 0.05; *** denotes significant correlations *p*-value < 0.001.

In patients, nonverbal social perception deficits failed to correlate with positive symptoms, negative symptoms (PANSS negative and BNSS), or overall symptom severity (PANSS total; see Figure 3). However, the BNSS subscales and the gesture expression item in patients indicated that nonverbal social perception deficits were linked to avolition, blunted affect and gesture expression, but not to anhedonia, asociality or alogia (see Table 3). In addition, no correlation was observed in either patients or controls between Mini-PONS and subjective negative symptoms (SNS; see table Figure 3).

**TABLE 3:** BNSS subscales/item correlations with PONS total score controlling for medication in patients.

	*r*	*p*
Anhedonia subscale	−0.1	0.574
Asociality subscale	−0.1	0.762
Avolition subscale	−0.3	0.045*
Blunted affect subscale	−0.3	0.031*
Alogia subscale	−0.2	0.188
Gesture expression item	0.5	0.004**

* denotes significant correlations p-value < 0.05; ** denotes a significant correlation p-value < 0.01. P-values marked with bold indicate statistically significant correlations.

**FIGURE 4:**
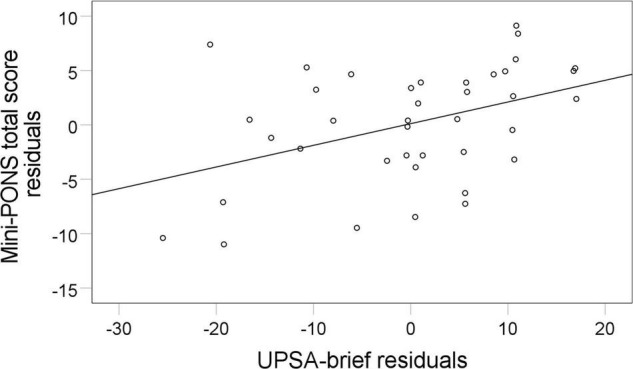
Association between nonverbal social perception and functional capacity. Mini-PONS, mini profile of nonverbal sensitivity; UPSA-brief, University of California San Diego performance-based assessment. To plot the partial correlation between Mini- PONS total score and UPSA-brief whilst controlling for medication (*r* = 0.42, *p* = 0.031), we used Mini-PONS total score residuals and UPSA-brief total score residuals.

**FIGURE 5:**
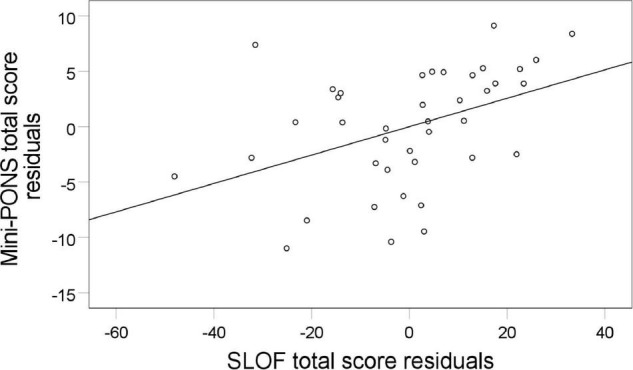
Association between nonverbal social perception and functional outcome. Mini-PONS, mini profile of nonverbal sensitivity; SLOF, specific levels of functioning scale. To plot the partial correlation between Mini-PONS total score and SLOF whilst controlling for medication (*r* = 0.41, *p* = 0.031), we used Mini-PONS total score residuals and SLOF residuals.

Poor nonverbal social perception highly correlated with limited functional capacity (UPSA-brief, see Figures 3, 4) and reduced functional outcome in patients (SLOF, see Figures 3, 5). While all symptom domains correlated with the rater-based SLOF total score, symptom domains failed to correlate with the performance-based UPSA-brief total score. When running correlations between Mini-PONS total score and all six SLOF subscales in patients, we observed that nonverbal social perception deficits are associated with impaired personal care skills, limited activities and reduced work skills, but not with physical functioning, poor interpersonal relationships and social acceptability (see Table 4).

**TABLE 4:** SLOF subscales comparison between schizophrenia patients and healthy controls; SLOF subscales correlation with Mini-PONS total score controlling for medication in schizophrenia patients.

	Comparison with healthy controls	Correlation with Mini-PONS in patients
Physical functioning subscale	*t* = −0.2; *p* = 0.855	*r* = 0.2; *p* = 0.149
Personal care skills subscale	*t* = 5.3; *p* < 0.001***	*r* = 0.5; *p* = 0.002**
Interpersonal relationships subscale	*t* = 9.0; *p* < 0.001***	*r* = 0.2; *p* = 0.144
Social acceptability subscale	*t* = 1.8; *p* = 0.086	*r* = 0.0; *p* = 0.951
Activities subscale	*t* = 6.6; *p* < 0.001***	*r* = 0.4; *p* = 0.008**
Work skills subscale	*t* = 11.0; *p* < 0.001***	*r* = 0.3; *p* = 0.026*

* denotes a significant correlation p-value < 0.05; ** denotes significant correlation p-value < 0.01; *** denotes significant differences p-value < 0.001. P-values marked with bold indicate statistically significant differences and correlations.

## Discussion

This study on nonverbal social perception deficits in schizophrenia patients had three main findings. First, we confirmed that schizophrenia patients perform worse in nonverbal social perception (Mini-PONS) compared to healthy controls (see Figure 1), especially when the task requires them to recognize emotional prosody (Mini-PONS voice recordings only and Mini-PONS voice recordings with facial expressions, see Figure 2). Second, as expected, in patients with schizophrenia, impaired nonverbal social perception was associated with limited functional capacity (UPSA-brief, see Figure 4) and poor functional outcome (SLOF, see Figure 5). On the SLOF subscales, poor nonverbal social perception correlated with poor personal care skills, limited activities and impaired work skills (see Table 4). Third, nonverbal social perception deficits were not linked to symptom domains (PANSS positive, PANSS negative, PANSS total, BNSS and SNS), with the exception of two BNSS subscales: avolition, blunted affect and one BNSS item: gesture expression (see Figure 3 and Table 3).

The first finding confirms that schizophrenia outpatients have nonverbal social perception deficits, which suggests that they have trouble recognizing nonverbal cues. This finding not only aligns with the well-established finding that patients with schizophrenia display difficulties at detecting emotions from facial expressions (Kohler et al., 2010; Olbert et al., 2013; Barkl et al., 2014), but also corroborates previous studies that used the same measure of nonverbal social perception (Monti and Fingeret, 1987; Bucci et al., 2008; Walther et al., 2016; Pinkham et al., 2017). Particularly, our findings demonstrate that schizophrenia patients have specific difficulties recognizing scenarios including voice recordings, while they performed similarly to controls on other scenarios, i.e., facial expressions only or body movements only. Hence, schizophrenia patients seem to have specific difficulties extracting emotional information from voice recordings, i.e., prosody. This is in contrast to previous papers reporting deficits in all Mini-PONS subscales (Toomey et al., 2002). Our findings also align with studies reporting that schizophrenia patients exhibit deficits in processing bimodal sensory information (Meiselman, 1973; Mussgay and Hertwig, 1990) and impairments in multisensory integration (de Jong et al., 2009; Tseng et al., 2015; Grohn et al., 2022), which suggest that individuals with schizophrenia have difficulties processing sensory signals from different modalities (e.g., visual and auditory modalities) of temporally and/or spatially coincident sources of information. However, in the current report we found schizophrenia patients to exhibit perceptual impairments in both unimodal and bimodal stimulus presentations. Similar to healthy controls, patients’ performance improved with bimodal presentation vs. unimodal presentation, however, the group difference remained. Collectively, these findings suggest that bolstering nonverbal information with bimodal stimuli, such as video clips, enhances understanding in patients, who nevertheless perform poorer than healthy controls.

Our second finding confirms and extends previous studies noting nonverbal social perception to be associated with both performance-based functional capacity (UPSA-brief) and observer-rated functional outcome (SLOF). These associations hold true even after controlling for medication dosage (chlorpromazine equivalent doses – mg/day) and correcting for multiple comparisons (FDR). While earlier studies also reported poor nonverbal social perception to be correlated with low functional outcomes in schizophrenia patients (Green and Horan, 2010; Walther et al., 2016; Pinkham et al., 2017), the present study extends these findings by exploring the correlations in distinct functional domains and functional capacity. Impaired personal care skills, limited activities and reduced work skills were correlated with impaired nonverbal social perception, while reduced physical functioning, interpersonal relationships and social acceptability were not.

Previous studies failed to find a significant correlation between nonverbal social perception deficits and symptoms (Toomey et al., 2002; Giannitelli et al., 2015; Walther et al., 2016). However, we have found that some negative symptom domains correlate significantly with these deficits, while others do not. The absence of correlation between nonverbal social perception deficits and symptoms may suggest that nonverbal social perception impairment is a core feature of schizophrenia, independent of current symptom severity, duration of illness and medication. This finding calls for further investigation linking nonverbal social perception with distinct symptoms domains. We extend previous reports by exploring associations within negative symptom domains. Here, we found that nonverbal social perception deficits correlated with some negative symptom domains (avolition, blunted affect), but not with others (anhedonia, asociality, alogia). Furthermore, the BNSS gesture expression item strongly correlated with nonverbal social perception, suggesting that patients rarely using gestures themselves also have difficulties understanding nonverbal social cues of their encounters. A related concept is the (dis-)embodiment perspective (Stanghellini, 2009; Walther et al., 2014; Tschacher et al., 2017), which implies that the relationship between cognitive-emotional processes and the body is altered in schizophrenia patients. Moreover, the finding of the current study corroborates previous reports of a generalized nonverbal communication deficit in schizophrenia (including gesture perception and production) (Walther et al., 2015, 2020b). Considering that gesture performance is highly correlated with functional outcome in schizophrenia (Walther et al., 2016), future work should test interventions alleviating nonverbal communication deficits in schizophrenia.

The strengths of our study are a homogeneous patient sample, a comprehensive assessment of nonverbal social perception, the focus on multiple domains of functional outcome and functional capacity. At the same time, our study is limited by the selection of stable, chronic outpatients, therefore, our results cannot be generalized to all patients with schizophrenia. Another limitation might be the absence of comprehensive neurocognitive assessments as cognitive deficits may have an effect on nonverbal social perception. We attempted to address this potential limitation by controlling for working memory in our main analyses. Furthermore, medication might also have an effect on our findings; however, we tried to tackle this problem by correcting for current medication dosage. Moreover, the Mini-PONS is only one of several potential tests of nonverbal social perception in schizophrenia and it takes some time to complete, but it offers sufficient internal consistency and retest-reliability (Pinkham et al., 2017). The assessment of the functional outcomes does not integrate information provided by caregivers or other informants (Harvey et al., 2019). However, we included the objective performance-based measure UPSA-brief to increase the reliability of our functional assessments. Finally, this cross-sectional study does not allow for inferences about causality. These issues will be addressed, once the interventional study is completed.

Future studies should determine whether specific interventions will alleviate nonverbal social perception deficits. Reducing nonverbal social perception deficits is considered to be important to improve social and community functioning in schizophrenia (Walther et al., 2016; Pinkham et al., 2017). Recently, studies using cognitive social remediation therapy (Kurtz and Richardson, 2012; Muller et al., 2014; Mueller and Roder, 2017; Bin Kitoko et al., 2020; Vita et al., 2021), non-invasive brain stimulation (Mehta et al., 2014; Walther et al., 2020a), as well as, virtual reality (Rus-Calafell et al., 2014; Torregrossa et al., 2018; Pavlidou and Walther, 2021) all show promising results in alleviating some of the social deficits schizophrenia patients’ experience. This holds true for the reduction of symptom severity and improvement of emotional processing, theory of mind, social functioning, as well as interpretation and use of nonverbal social cues (i.e., gesture performance).

## Conclusion

Schizophrenia outpatients exhibit nonverbal social perception deficits, which are linked to their ability to function in everyday life. Nonverbal social perception deficits are associated with some negative symptoms (avolition, blunted affect, and item gesture expression) and with multiple functional domains (impaired personal care skills, activities and work skills). These findings suggest that the difficulty to decode nonverbal cues in schizophrenia patients is key to function in daily life. Future studies should investigate whether interventions designed to improve nonverbal social perception deficits could alleviate negative symptoms and improve overall functioning.

## Data Availability Statement

The raw data supporting the conclusions of this article will be made available by the authors, without undue reservation.

## Ethics Statement

The studies involving human participants were reviewed and approved by Kantonale Ethikkommission Bern (KEK). The participants provided their written informed consent to participate in this study.

## Author Contributions

VC recruited participants, conducted assessments, analyzed the data and drafted the manuscript. AP supervised and contributed to the statistical analyses. LM carried out clinical assessments. SK recruited participants and conducted assessments. SW designed the study, obtained funding, wrote the protocol, and supervised assessments. All authors discussed the findings and critically revised the manuscript.

## Conflict of Interest

SW received honoraria from Janssen, Lundbeck, Mepha, Neurolite, and Sunovion. The remaining authors declare that the research was conducted in the absence of any commercial or financial relationships that could be construed as a potential conflict of interest.

## Publisher’s Note

All claims expressed in this article are solely those of the authors and do not necessarily represent those of their affiliated organizations, or those of the publisher, the editors and the reviewers. Any product that may be evaluated in this article, or claim that may be made by its manufacturer, is not guaranteed or endorsed by the publisher.
